# Nanopores: maltoporin channel as a sensor for maltodextrin and lambda-phage

**DOI:** 10.1186/1477-3155-3-3

**Published:** 2005-03-02

**Authors:** E Berkane, F Orlik, A Charbit, C Danelon, D Fournier, R Benz, M Winterhalter

**Affiliations:** 1 Institut Pharmacologie & Biologie Structurale-CNRS UMR5089, 205, rte de Narbonne, F-31077 Toulouse, France; 2 Lehrstuhl für Biotechnologie, Biozentrum, Am Hubland, D-97074 Würzburg, Germany; 3 Inserm U-570, CHU Necker-Enfants Malades, 156, rue de Vaugirard, F- 75730 Paris Cedex 15, France; 4 International University Bremen, School of Engineering and Science, D-28727 Bremen, Germany

**Keywords:** Single molecule detection, Nanobiotechnology, Electrophysiology, Nanopore concept, porin

## Abstract

**Background:**

To harvest nutrition from the outside bacteria e.g. *E. coli *developed in the outer cell wall a number of sophisticated channels called porins. One of them, maltoporin, is a passive specific channel for the maltodextrin uptake. This channel was also named LamB as the bacterial virus phage Lambda mis-uses this channel to recognise the bacteria. The first step is a reversible binding followed after a lag phase by DNA injection. To date little is known about the binding capacity and less on the DNA injection mechanism. To elucidate the mechanism and to show the sensitivity of our method we reconstituted maltoporin in planar lipid membranes. Application of an external transmembrane electric field causes an ion current across the channel. Maltoporin channel diameter is around a few Angstroem. At this size the ion current is extremely sensitive to any modification of the channels surface. Protein conformational changes, substrate binding etc will cause fluctuations reflecting the molecular interactions with the channel wall. The recent improvement in ion current fluctuation analysis allows now studying the interaction of solutes with the channel on a single molecular level.

**Results:**

We could demonstrate the asymmetry of the bacterial phage Lambda binding to its natural receptor maltoporin.

**Conclusion:**

We suggest that this type of measurement can be used as a new type of biosensors.

Nature created and optimized proteins for specific tasks which makes them often interesting in material science. For example, membrane transporters could control the permeability of artificial nanometer sized container. A typical application could be to control the enzymatic activity in a liposome [1]. Another possible application is to reconstitute channels into planar lipid bilayer and use time dependent conductance as a signal [2,3]. Application of an external electric field drives the ions through the nano (and subnano) meter sized channel. Any larger molecule that diffuses into and temporarily sticks to the channel interior will cause typical fluctuations of the ion current which allow to conclude on its mode of translocation. Such studies were used to follow sugar translocation through maltoporin [4]. Similar types of measurements were done to investigate the translocation of antibiotics like ampicillin [5]. Subtle changes in the channel size or small conformational changes can be recorded and this technique could be developed towards an instrument to probe very soft forces.

Porins are attractive candidates for applications because they are very stable. Moreover, recombinant technology permits production of porins in *E. coli *with high yields [6]. A third advantage is the availability of the high resolution 3-D crystal structure showing details of substrate binding sites which facilitates enormously a rational engineering of modified proteins.

The outer cell wall of Gram-negative bacteria from *E. coli *is fairly permeable to smaller solutes below a molecular weight of about 400 Da [6]. Such substances can freely permeate under a concentration gradient through general diffusion porins in the outer cell wall. Under stress, e.g. in case of lack of nutrition, the pure diffusion process is too slow and the bacteria need to improve the efficiency of the translocation. For those cases, nature has created a series of rather specific and highly sophisticated membrane channels. The most extensively studied examples of specific porins are the maltooligosaccharide-specific channel Maltoporin of *E. coli *[4,7,8]. Maltoporin forms ion-conducting channels when reconstituted into lipid bilayers [9,10]. The 3D structure of Maltoporin revealed that the monomer of Maltoporin of *E. coli *consists of an 18 stranded β-barrel with short turns at the periplasmic side and large irregular loops at the outside of the cell [11].

The bacteriophage Lambda is a virus recognizing Maltoporin at the outer cell surface [12]. In absence of this membrane channel, phage Lambda does not recognize the bacteria. Or, even minor mutations allow the bacteria to defend themselves against virus attacks. The virus itself can, in turn mutate to restore binding ability. According to the high resolution X-ray structure the water filled channel is far too small to permit the translocation of the double strain DNA (about 20 Å) [11]. The infection mechanism thus must involve one of the following processes: Phage binding will cause a strong conformational change within the Maltoporin or, after binding the phage releases a DNA translocation machinery to bring its DNA across the hydrophobic membrane. To date none of these intermediate steps has been observed so far and the underlying process remains unclear. Recently, gpJ, a protein in the phage terminal was identified to be involved in the Maltoporin recognition process [13].

A typical set-up for conductance measurements is shown in figure 1. The measurement cell consists of two chambers separated by a hole (less than 0.1 mm diameter) in a thin poly(tetrafluorethylene) film sandwiched between two half-cells made of Teflon (Goodfellow, Cambridge, UK). Prior to each measurement this hole has to be pretreated to render it lipophilic by coating it with a hexadecane/hexane (1:100 v:v) droplet. After allowing for hexane evaporation, each chamber is filled with 1.5 ml buffer (for example, 1 M KCl, unbuffered, about pH 6). Black lipid bilayers were formed according to the classical Montal-Mueller technique by spreading lipids in hexane/chloroform (9:1) across the aqueous buffer [14]. For sake of stability we used diphytanoyl-phosphocholine (DphPC, Avanti Polar Lipids). After 20 min allowing for evaporation, the buffer level is lowered below the hole level and rose again. Typically after the first or second trial a stable unilamellar membrane is formed. In order to insert single porin trimers within reasonable time, but to avoid insertion of their multiples, a careful balance between the concentration of the protein solution, detergent concentration and buffer volume has to be found. One single porin trimer has to find the membrane and to insert while all others must be inactivated, e.g. by precipitation. Maltoporin from the stock (1 mg/ml in 1% OPOE) was diluted 10^2^-10^5 ^times in the buffer containing 1% OPOE. From our own experience in our laboratory the insertion was optimal if smallest amounts (less than 1 μl) were injected. In a second measurement we used painted membranes as described previously [15]. Here the Teflon chamber consists of a larger hole (diameter 800 μm and larger). Membranes were formed by painting 1 μl of a 1% solution of DphPC in n-decane across the hole. This type of membrane facilitates multichannel insertion.

**Figure 1:**
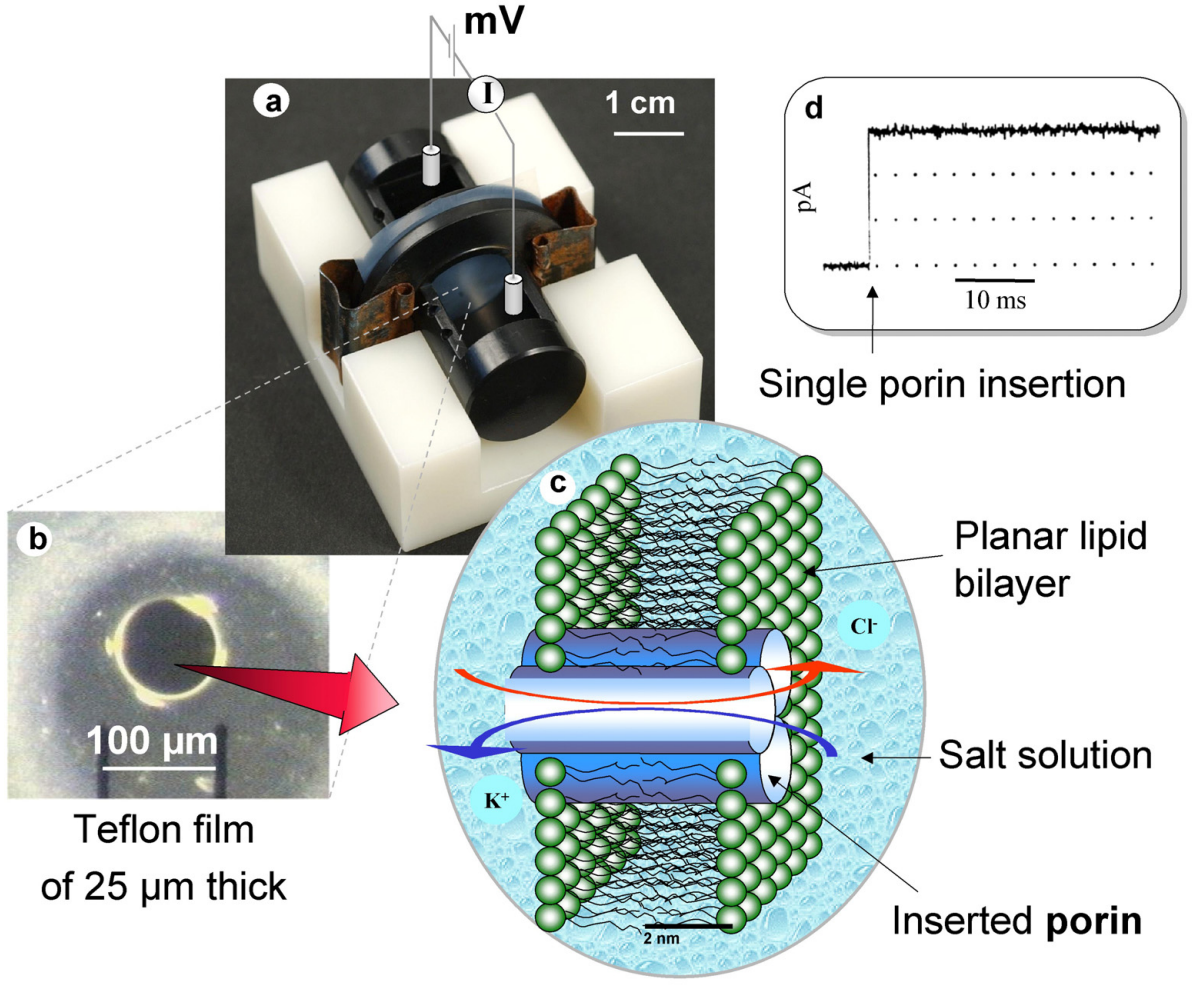
Schematic representation of a typical planar bilayer set-up for ion current recording. 1.a) Two half cells made of Delrine separated by a 25 μm Teflon foil with a hole in the center. Both parts are clamped together. 1.b) Below a microscope picture of the Teflon septum containing a hole. 1.c) Schema of a lipid bilayer with a reconstituted trimeric porin. The Cl^- ^ions are attracted to the positive electrode and K^+ ^to the negative one. Ions are permeating the channel in the MHz range which is beyond the current time resolution. 1.d) The insertion of a single channel will give raise to a jump in conductance. Any objects diffusing in the channel may reduce the permeation time of ions and may be detected either in conductance fluctuations or an averaged reduced conductance.

Membrane current was measured via homemade Ag/AgCl electrodes. One electrode was used as ground and the other connected to the headstage of an Axopatch 200B amplifier (Axon Instruments, USA), allowing the application of adjustable potentials (typically, 100 mV) across the membrane. A similar set-up was used in the second measurement.

We recently investigated the sugar penetration on a single molecular level [4]. We were able to reconstitute a single Maltoporin trimer into the lipid bilayer. Addition of sugar into the bulk phase resulted in a blocking of the channel in a concentration dependent manner. At low sugar concentration individual closure of the channel could be observed. Maltohexaose induces higher frequencies of closure and longer closing times than a smaller sugar like maltose. The analysis of the time-resolved conductance as a function of sugar concentration yielded the binding constant as well as the "on" and "off" rates for the sugar binding. Here we used a modified sugar through covalent binding of an ANDS (3-amino-naphtalene-2,7-disulfonic acid) molecule to the reducing end of a Maltoheptaose as schematically shown in fig. 2A (for details, see [16]). The crystal structure suggests that the maltose molecule enter the channel only with the nonreducing end from the outside (or reducing end from the periplasmic side). Subsequently this molecule can only enter from the cis-side in our setup. In fig. 2B we see that addition on the periplasmic side (trans side) inhibit the entry whereas addition to the outer side (cis side) caused blocking. A good control experiment in order to test the activity is to add unmodified sugar molecules to the previous experiment. In fig. 2C we clearly observe the ability to translocate unmodified sugars. Addition of small amounts of unmodified sugar to the trans-side caused the expected number of events. Further addition of unmodified sugar to the opposite site enhances the sugar induced blocking. These data can be used for a fundamental analysis to probe e.g. the individual energy barrier and it seems that nature has optimized this channel to have the best turnover number. On the other hand these channels can potentially serve to discriminate sucrose from maltose.

**Figure 2:**
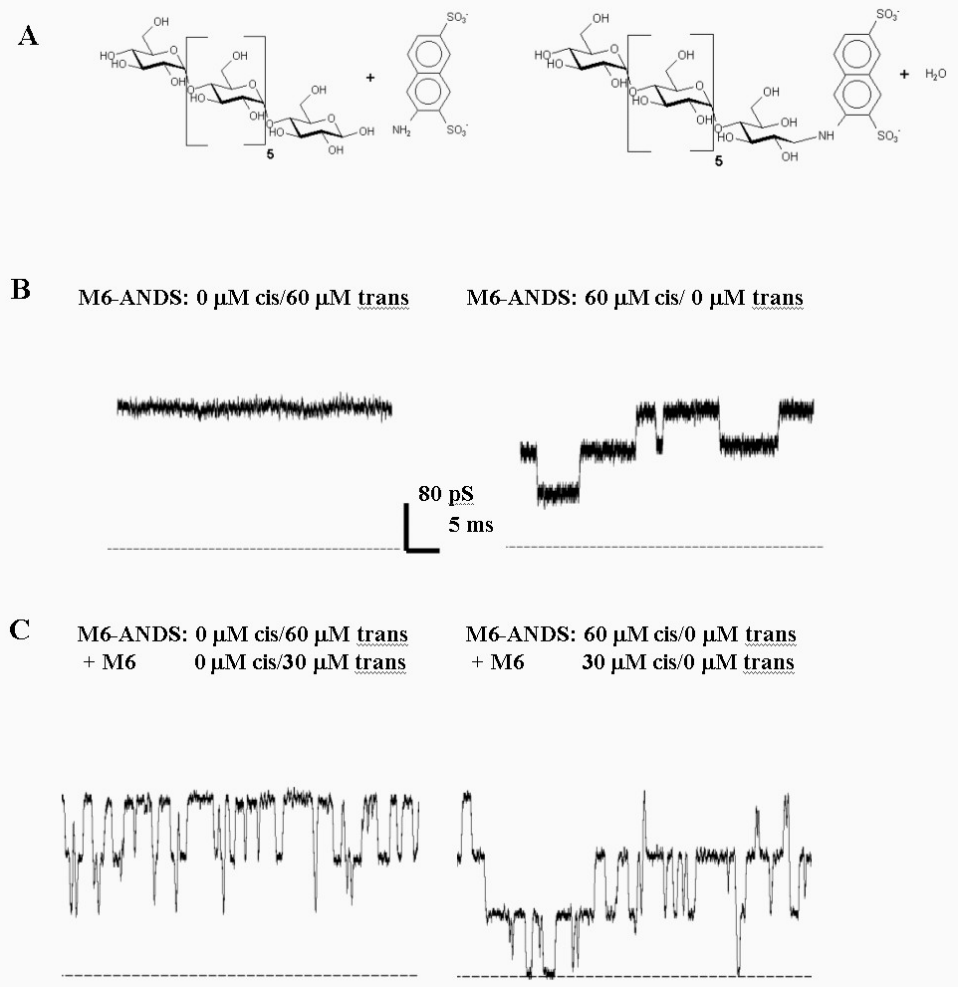
Typical recordings of ion current through a single Maltoporin trimer in presence of modified maltohexaose (see [16] for details). (**A**) Shows the unmodified maltohexaose and on the right hand side the modified sugar molecule. We designed this molecule according the crystal structure to guarantee the low penetration ability from one side. (**B**) M6-ANDS was added to *trans *(left) and then to *cis *(right). Sugar analogue modulates ion current only to the cis-side, the side of Maltoporin addition. The average residence time is 5.0 ms. (**C**) First, M6-ANDS was injected to the trans-side and no variation in the ion current occurs. As control, maltohexaose was added to the same side (left). The natural substrate is translocated demonstrating that it enters the channel from *trans *with the reducing end first. Then, M6-ANDS was added additionally to the cis-side (right) generating long current interruptions superimposed to maltohexaose blockade events seen in the figure of the left side. The dashed lines corresponding to zero current. Membrane bathing solution was 1 M KCl, 10 mM Tris, 1 mM CaCl_2_, pH 7.4, the applied voltage was + 150 mV.

In a second series of experiments we were interested to probe for Lambda phage binding. In principle this should be possible despite the enormous size (about 100 nm size in comparison to 4 nm sized channels). However in a preliminary step we have produced larger quantities of the phage endterminal protein gpJ fused to Maltose Binding Protein (MBP). We reconstituted a larger number of maltoporin in solvent containing membranes and titrated small quantities of the fusion construct MBP-gpJ. We know from the experiments described above that most of the channels are oriented the same direction during the reconstitution. In fig. 3 we show a first result that titration of gpJ to the opposite side of protein addition had no effect. In contrast, addition of gpJ to the side of porin addition caused rapid blocking of the channel. This observation suggest that the porin inserts with the short turns first and that the protein part exposed to the extracellular side is naturally accessible to Lambda phages. These first results are promising and we currently work on improving the resolution. Here we have to note that this observation is in clear contrast by a report on phage lambda binding in a multichannel preparation [17]. The origin of this discrepancy might be simultaneous multiple insertion. Our observation here is in agreement with other reports showing the same orientation [4,5,18]. However, reason why porins inserts in artificial membranes differently than in natural ones remains unclear. One may speculate that the strong asymmetry of natural membranes or unknown chaperons will facilitate the entry with the long loops first.

**Figure 3:**
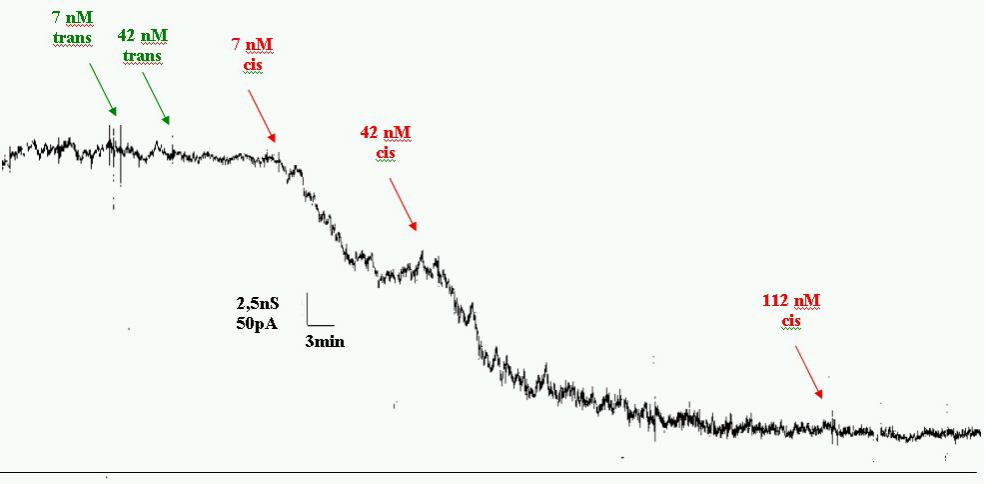
Here we show the ability to recognize bacterial phage Lambda by blocking the ion conductance through the natural receptor Maltoporin. We first reconstituted about 300 Maltoporin channel in a solvent containing planar lipid bilayer. This leads to a stable conductance after about 30 min with no further protein insertion. Titration of 7 and 42 nM of the fusion protein MBP-gpJ from the bacterial virus Lambda to the compartment corresponding the intracellular side of the channel showed no effect. However, titration to the opposite side corresponding to the extracellular side caused a significant reduction of the ion conductance. Membrane bathing solution was unbuffered 1 M KCl giving a pH of about 6. The applied voltage was + 20 mV.

Sensing with membrane channel is a new way in screening for solute molecules and several promising examples are already shown [2,3,16,19,20]. The actual bottleneck is the complexity in membrane channel assembly. However, the current development in automatized patch-clamping will open a wide range of possibilities [21,22]. We plan to reduce the volume on each side of the membrane and the size of the lipid patch. We currently work with pore diameters of about 1 μm with less background capacitance and thus a better time resolution and to simplify the channel assembly.
